# Recommended recumbency to avoid insertional complications during small-bore wire-guided thoracostomy tube placement in cats—a cadaver study

**DOI:** 10.1186/s12917-024-04301-7

**Published:** 2024-10-01

**Authors:** Desiree Siegelmayer, Eva Schnabl-Feichter, Alexander Tichy, Gabriele Gradner, Brigitte Degasperi, Lea Liehmann

**Affiliations:** 1 University Clinic for Companion Animals of Vienna, Veterinärplatz 1, Vienna, 1210 Austria; 2 Department of Biomedical Science, Veterinärplatz 1, Vienna, 1210 Austria; 3 Tierarztpraxis am Stadtpark GmbH, Reisnerstrasse 7/1, Vienna, A-1030 Austria

**Keywords:** Chest tube, Pleural effusion, Cat, Cadaveric study, Small-bore thoracostomy tubes, Wire-guided thoracostomy tubes, Seldinger technique

## Abstract

**Background:**

Small-bore wire-guided thoracostomy tubes (SBWGTT) are commonly used in cats to manage pleural disease and generally have a low complication rate. Our study aimed to explore the correlation between recumbency of cats, placement method, and the occurrence of insertional complications to identify risk factors during SBWGTT placement.

In this experimental cadaveric study, SBWGTT placement using a modified Seldinger technique was conducted in 24 feline cadavers. Cats, euthanized for reasons unrelated to the study, were randomly assigned to pleural effusion (EFF; *n* = 12) and pneumothorax (PNEU; *n* = 12) groups. Each cadaver was intubated and ventilated with a peak inspiratory pressure (PIP) of 10 mmHg, and sterile saline or air was instilled into the thorax over a 5 mm thoracoscopic trocar in the fourth intercostal space (ICS). Instillation was stopped when the lateral thoracic wall to lung distance (TWLD) reached 10 to 12 mm, measured with ultrasound in the favorable position. Sternal recumbency was the favorable position for the EFF group, and lateral recumbency for the PNEU group. Following the placement of the first SBWGTT in each group, the cadavers were positioned unfavorably (lateral recumbency for EFF group, sternal recumbency for PNEU group), and a second drain was introduced contralaterally. A bilateral 8th ICS thoracotomy was then performed to visually assess intrathoracic structures and drain integrity.

A binary logistic regression mixed model was conducted to determine interaction between the induced condition and body position.

**Results:**

A total of 48 SBWGTTs were placed, with complications observed in 33.3% (8/24) of cases. Five of these were major complications consisting of lung lacerations. Complications were more common in the unfavorable position, accounting for 75% of cases, although this result was not statistically significant. The odds of complication rates were > 70% in the unfavorable position and decreased with an increase in TWLD (< 30%).

**Conclusion:**

Complications associated with SBWGTT placement are influenced by recumbency, although the data did not reach statistical significance. Placing cats in lateral recumbency for pneumothorax treatment and sternal recumbency for pleural effusion treatment may reduce insertional complications.

## Background

Tube thoracostomy is a commonly performed procedure in both human and veterinary medicine to evacuate gas or fluid from the pleural space, either following pleural effusion or pneumothorax alone or as part of thoracic surgical procedures [1–6].

While thoracocentesis is primarily used for diagnostic sampling and patient stabilization, thoracostomy tube (TT) placement is indicated in patients with recurrent pneumothorax, tension pneumothorax, rapidly accumulating pleural effusion, pyothorax, and following thoracotomy [7].

Large-bore thoracostomy tubes (LBTT) have traditionally been used for evacuation of large amounts of non-viscous, viscous and fibrinous/flocculent fluids [8, 9], while small-bore thoracostomy tubes (SBTT) have been introduced for evacuating air and non-viscous fluids [10–12]. Differentiation is also made based on the method of insertion, which can be through a trocar technique, blunt dissection into the pleura (mini-thoracotomy), or a modified Seldinger guide-wire technique [7, 11, 13–16]. The most commonly used drainage systems in veterinary patients include traditional large-bore trocar tubes (TRO), small-bore wire-guided thoracostomy tubes (SBWGTT), and Jackson Pratt thoracostomy drains (JP) [11, 13, 17, 18], with the latter used exclusively intraoperatively during thoracic surgery to provide relieve of postoperative pneumothorax or pleural effusions [17].

In both human and in veterinary medicine, large-bore trocar drains have become less popular due to their high complication rate [3, 15, 19, 20]. Reported insertional complication rates associated with LBTT range from 3 to 35% in humans [2, 20–24] and up to 58% in veterinary patients [14, 15, 25–28]. Trocar drains as well as the “mini-thoracotomy” technique typically require general anesthesia, which may not be suitable for cardiovascular unstable or dyspneic animals [7, 25, 26, 29].

The Seldinger technique has gained popularity in both human medicine [30, 31] and veterinary medicine over the past decade due to its ease of use and patient comfort [11, 17, 32]. It offers a simpler alternative for chest drain placement in both small animals and humans [11, 31–33]. SBWGTT are recommended for treating pneumothorax and malignant effusion in humans due to fewer insertional and infectious complications, as well as better patient tolerance [34–37].

Valtolina et al. (2009) first described insertion of SBWGTT using the modified Seldinger technique in dogs and cats, noting minimal insertional complications and quick thoracic evacuation [11]. Del Magno et al. (2020) also presumed SBWGTT to be a safe option for managing feline pyothorax with lower complication rates [11, 33].

Despite the benefits of thoracostomy tube drainage, various complications can arise [2]. In human medicine, tube thoracostomy is linked with significant morbidity [21]. Reported complications encompass incorrect placement (e.g. entering too far cranially or insufficiently into the thorax), failure to evacuate the pleural space (obstruction of the tube with fibrinous debris, coiling or kinking of the tube), unresolved pneumothorax (persistent air leakage or residual pneumothorax), persistent effusion, infection, air and/or fluid leakage, stoma site discharge, stomal infection, empyema, lung tissue irritation, re-expansion pulmonary edema, phrenic nerve irritation or neuropraxia, injury to the sympathetic chain, injuries to intercostal or intrathoracic vessels, injuries of the diaphragm or the subdiaphragmatic viscera, premature accidental tube removal or dislodgement, and inadvertent loss of negative intrathoracic pressure resulting in pneumothorax [7, 17, 22, 28, 38–40].

Merca et al. (2021) conducted a retrospective evaluation of the complication rates of four types of thoracostomy tubes in 201 dogs and 139 cats, with a total of 455 drains placed. They established a classification scheme for complications, which included positional (33.1%), infective/immunologic (19.4%), instructional/educational/equipment related (19.1%), removal (8.2%), insertional (7%), and self-mutilation-related (5%) issues. Over-the-wire and blunt and sharp wide-bore tubes were most associated with positional, infective or equipment-related complications [41]. Thus, thoracostomy tube placement, although common in human medicine and veterinary medicine, carries the potential for significant morbidity and mortality [2, 21, 38].

A retrospective study by Boullhesen Williams et al. in 2022 shared concerns regarding the safety of SBWGTT, reporting a substantial complication rate of 32%, with 21.7% technical and 14.1% insertional issues [18].

The aim of our study was to investigate the correlation between patients’ recumbency, placement method, and the occurrence of insertional complications in order to determine factors increasing safety and minimizing risk of complications during thoracostomy tube placement with a SBWGTT.^1^

We hypothesized that for the treatment of pneumothorax, SBWGTT produces fewer insertional complications in lateral recumbency compared to sternal recumbency, and vice versa for the treatment of fluid-filled thoraces.

To the author’s knowledge, no studies have been published comparing different recumbencies for thoracostomy tube placement using small-bore wire-guided thoracostomy tubes in different pleural cavity conditions.

## Material and methods

### Study design

This ex vivo study comprised a pilot study and a main experimental study. During the pilot study, we assessed the average penetration depth during thoracic needle puncture using an ultrasound probe and machine.^2^ Based on this measurement, the amount of air or liquid to be instilled into the thoracic cavity was determined to prevent the needle tip of the SBWGTT from contacting the lung during placement for the main study.

A total of 30 feline cadavers were collected for the entire study, with six cats allocated to the pilot study and 24 cats to the main study. All cats were humanely euthanized for reasons unrelated to this study. Patients were euthanized by the intravenous injection of sodium pentobarbital and were previously premedicated with either methadone, buprenorphine, or dexmedetomidine in combination with propofol, depending on the clinician’s preference and patient’s condition.

For each cadaver, medical records were reviewed for information regarding signalment, medical history, examination findings, diagnostic imaging, if available, and reason for euthanasia. Inclusion criteria were fresh feline cadavers (< 48 h) of variable sex and breed, with a mean body weight (BW) of 2.5–5 kg, confirmed by a veterinarian to have no signs of respiratory disease based on medical history and physical examination. Exclusion criteria were patients weighing less than 2.5 kg BW and younger than one year. There was no age limitation for older cats. Patients were excluded if they had any pneumopathies (lung edema, feline asthma, pneumonia, or others), signs of pleural effusion or pneumothorax, or respiratory distress prior to euthanasia. Cats that underwent cardiopulmonary resuscitation before euthanasia were also excluded, as they were assumed to have possibly impaired lungs and ribs. Cats with feline aortic thromboembolism were included if they exhibited no clinical signs of a lung edema or pleural effusion, as verified by ultrasound.

Cadavers were either preserved in a cooler at 9.6 °C until the trial, rewarmed to room temperature prior to the experiment, or utilized immediately after euthanasia. Each trial was performed at room temperature. No macroscopic evidence of disease of the thoracic wall was noted during specimen preparation.

### Pilot study

#### Patients

Patients were collected between October 1st to 31st, 2022 at the University Clinic for Companion Animals in Vienna.

A total of six cat cadavers were used for the pilot study and divided into two groups: a young group aged 1–3 years to mimic patients with pneumothorax or pleural effusion due to trauma like high rise syndrome (*n* = 3), and an older group consisting of three cats older than 9 years (*n* = 3) to imitate patients with pleural effusion like chyle or malignant effusion.

#### Procedure

Thoraces of the six cats were clipped bilaterally, and the third to the twelfth intercostal spaces were marked in the mid-thorax. Cannulas were inserted in the sixth, seventh, eighth, and ninth intercostal space (ICS) through the intercostal muscles until entrance into the pleural cavity was felt. This procedure was performed in lateral recumbency, with puncture of the left and right hemithorax randomly assigned. In total, four punctures per side per feline cadaver per person were carried out.

Two investigators conducted the experiment: a boarded ECVS diplomate and a less experienced veterinarian. The decision of who was puncturing dorsally to the marked site was also block randomized. An ultrasound probe and machine^2^ were used to determine the entrance depth of the needle.

Inter- and intraobserver variabilities were calculated, as well as mean and standard deviations. The measurements obtained from the pilot study were then employed to refine the procedure for the main study, calculating a value (calculated value of the pilot study; MP) to ensure that the needle tip of the SBWGTT avoids contacting the lung during placement.

### Main study

#### Animals

Patients were collected between March and October of 2023 at the University Clinic for Companion Animals in Vienna. In total, 24 feline cadavers were sampled, all meeting the inclusion criteria and were used for data collection.

#### Procedure

In 24 cats, a 14-gauge small-bore wire-guided chest drain (MILA International Inc.®^1^; SBWGTT) was placed using a modified Seldinger technique, as described by Valtolina and Adamantos (2009) [11, 16].

All feline cadavers were intubated and ventilated with an appropriately sized endotracheal tube connected to an anesthetic machine with a mechanical ventilator. The lungs were mechanically ventilated with a peak inspiratory pressure (PIP) of 10 mmHg [42].

The cohort was separated in two randomly assigned groups in matched pairs: in twelve cats, iatrogenic pneumothorax was created by instilling air into the thoracic cavity (PNEU *n* = 12) and iatrogenic pleural effusion was induced by instilling isotone saline solution into the thorax in the other twelve cats (EFF *n* = 12). The starting side was randomly assigned to be the left or right hemithorax (50:50 per group, *n* = 6). Patient recumbency was chosen to start in the favorable position: sternal recumbency for the EFF group and lateral recumbency in the PNEU group.

To prepare for these conditions, the thorax was clipped, and a 5 mm port (Dilating Tip Trocar with Stability Sleeve^3^) was inserted into the 4th intercostal space. The thoracic cavity was then insufflated with either isotone saline solution or air until the distance from the lateral thoracic wall to the underlying lung (TWLD) equaled the previously determined penetration depth in the favorable recumbency (1.1–1.2 cm; numbers from pilot study). The distance was measured ultrasonographically^2^.

A stab incision was made at the 8th intercostal space with a no. 11 blade. For cases in the PNEU group, this was located between the middle and the dorsal third, and for cats in the EFF group, the incision was made between the middle and the ventral third of the thoracic wall. The introducer catheter was inserted and advanced into the pleural space at the cranial edge of the rib to minimize the risk of injuring the neurovascular bundle, situated at the caudal aspect of the rib. The stylet was removed, and a 0.035-inch guidewire (J-wire) was threaded through the catheter and advanced cranioventrally approximately 12 to 20 cm or until resistance was encountered. The catheter was withdrawn, and a dilator was slid over the J-wire to enlarge the orifice. After removing the dilator, the chest drain was advanced over the wire into the thoracic cavity. The wire was then removed, and the drain capped [11].

During the procedure, the thoracic cavity was completely sealed to prevent any leakage of air or liquids. After placement of the first drain, cats were placed in the unfavorable position: lateral recumbency for the EFF group and sternal recumbency for the PNEU group. TWLD was then measured again ultrasonographically^2^ as described before. A second SBWGTT (MILA International Inc.®^1^) was inserted in the same manner as the first one.

Thoracostomy tubes were placed by a ECVS diplomate (LL, BD, GG). A lateral thoracotomy was performed in the 8th intercostal space to explore the thoracic cavity for possible macroscopic changes associated with the thoracostomy tube placement. Macroscopic traumatic injuries to lungs, heart, vessels, nerves, diaphragm, and subdiaphragmatic viscera, as well as the integrity of the drains and connecting devices, were noted and statistically compared.

#### Evaluation

Complications were systemically classified into minor and major complications. Major complications were characterized by their potential to require surgical intervention in the live animal, such as lung lobe laceration. On the other hand, minor complications encompassed issues that did not have serious consequences for the patient but added complexity to the procedure, such as kinking of the wire. Noteworthy abnormalities that had no bearing on the well-being of the patient were documented separately, such as placement deviation (deviation from the optimal placement and position of the drain without serious consequences).

### Statistics

All statistical analysis were conducted using statistical software “IBM SPSS v29”. Effects on the presence or absence of damage were evaluated via a binary logistic regression mixed model in R, treating the damage as a binary response (absence = 0, presence = 1). The interaction between the induced condition and body position was treated as a fixed effect. The individual ID was treated as a random effect to account for the covariance structure in our data, as each individual was examined twice. Multicolinearity was assessed with the variance inflation factor (package *car*, function *vif*) [43]. Significance was declared at an alpha cut-off of 5% (*P*-value < 0.05 was considered significant). Since there was no prior knowledge of the expected presence or absence of damage in such an experimental setup, a formal sample size calculation was not feasible. Instead, we aimed for at least 10 ‘events per variable’ of the binary logistic regression mixed model [44–46]. We concluded that at least 24 animals were needed for accurate coefficient estimation in the model. For descriptive purposes, distributed data were recorded as a mean ± standard deviation or medians and ranges, depending on data distribution.

## Results

### Pilot study

A total of 96 punctures were performed in six feline cadavers. 48 punctures were done per person, which equals eight punctures per cat per investigator.

#### Animals

All six cats were domestic shorthairs, with three neutered females, two neutered males and one intact female. The median age of the young group was 1 year and 6 months, and of the older group, 14 years and 1 month. The median BW was 3.05 kg (2.8 to 4.0 kg BW). The underlying disease leading to euthanasia of the cats were multiple jaw fractures after trauma (*n* = 2), azotemia (*n* = 2), vestibular syndrome and anemia (*n* = 1), and treatment-resistant anorexia (*n* = 1).

#### Measurements

Overall median entrance depth was 0.8 cm, with a standard deviation of 0.3 cm (0.18 – 1.69 cm). The statistically calculated measurement for the ideal TWLD was 1.1 – 1.2 cm (MP), determined using the upper bounds of the 90% confidence interval of the mean differences. This value guided the main study procedure to ensure that the needle tip of the SBWGTT avoided contacting the lung during placement.

### Main study

A total of 48 drains were placed. The thorax of twelve cats was instilled with air (group PNEU) and water (group EFF) respectively. Group EFF was started in sternal recumbency, and group PNEU in lateral recumbency.

#### Animals

The breeds were distributed as follows (Table 1): domestic shorthair cats (*n* = 20), British shorthair cats (*n* = 2), domestic longhair cat (*n* = 1) and Carthusian cat (*n* = 1). Of these, 13/24 were female neutered, 9/24 neutered males, and 2/24 intact female. The median age was 12.9 years (median: 155 months; 36 – 288 months, 3 to 24 years) with a median BW of 3.8 kg (2.6 kg to 5.0 kg BW). The underlying disease causal for euthanasia of the cats were feline aortic thrombosis (*n* = 6), chronic kidney disease (*n* = 5), presentation in a clinically severe condition (*n* = 4), neurological signs (*n* = 4), including paraplegia in two cats, seizures in another, and suspicion of cerebral lesion in the other patient; anemia due to feline leukemia virus infection (FeLV) (*n* = 1), ascites (*n* = 1), gastric lymphoma (*n* = 1), multiple pelvic fractures (*n* = 1) and nasal neoplasia (*n* = 1).

**Table 1:** Information on signalment and cause of death of the cadavers used in the main study

	**Age**	**Body weight (kg)**	**Sex**	**Breed**	**Cause of death**
Cat 1	18 y 2 mo	4	fn	Carthusian	Severe neurologic signs
Cat 2	3 y	2,8	mn	DSH	Feline aortic thrombosis
Cat 3	10 y 4 mo	4,3	mn	DSH	Ascites of unknown origin
Cat 4	7 y 2 mo	4,8	mn	DSH	Shock (Suspicion: heart disease)
Cat 5	11 y	3,2	fn	DSH	Feline aortic thrombosis
Cat 6	14 y 9 mo	4,3	mn	DSH	Feline aortic thrombosis
Cat 7	10 y	2,6	fn	BSH	Severe anemia (Suspicion: feline leukosis)
Cat 8	12 y	3,1	fn	DLH	Nasal tumor
Cat 9	16 y	3,1	mn	DSH	Renal insufficiency
Cat 10	15 y	3,6	fn	DSH	Paraplegia, methemoglobulinemia
Cat 11	5 y 11 mo	3,7	fn	DSH	Feline aortic thrombosis
Cat 12	15 y 1 mo	3,8	f	DSH	Paraplegia
Cat 13	13 y 10 mo	4,6	mn	DSH	Gastric lymphoma
Cat 14	8 y	5	fn	DSH	Multiple pelvic fractures
Cat 15	8 y 2 mo	5	mn	DSH	Renal insufficiency
Cat 16	20 y	3,8	fn	DSH	Bad clinical condition
Cat 17	11 y 6 mo	3,9	fn	DSH	Feline aortic thrombosis
Cat 18	4 y 9 mo	4,6	fn	BSH	Renal insufficiency
Cat 19	14 y 10 mo	4,3	mn	DSH	Renal insufficiency
Cat 20	7 y	4,3	mn	DSH	Feline aortic thrombosis
Cat 21	16 y	2,6	fn	DSH	Renal insufficiency
Cat 22	20 y	3,1	fn	DSH	Bad clinical condition
Cat 23	17 y	2,7	f	DSH	Bad clinical condition
Cat 24	24 y	5	fn	DSH	Seizures

*DSH* Domestic shorthair, *DLH* Domestic longhair, *BSH* British shorthair, *EFF* pleural effusion, *PNEU* pneumothorax

#### Measurements

All cats were intubated, and the lungs ventilated using a peak inspiratory pressure (PIP) of 10 mmHg. The respiratory rate was 12 breaths per minute (10 – 16 breaths per minute, depending on body weight). In the favorable position, the achieved median TWLD was 1.16 cm (1.07 – 1.24 cm; SD 0.06 cm) for the EFF group and 1.15 cm (1.1 – 1.19 cm; SD 0.03 cm) for the PNEU group (Table 2).

**Table 2:** Measurements in favorable (1. TWLD) and in unfavorable (2. TWLD) recumbency, along with a list of complications observed during this study

	**Condition**	**1. TWLD (cm)**	**2. TWLD (cm)**	**Abnormalities**	**Complications**		**Comment**
Cat 1	EFF	1,1	0,57	Yes	No		
Cat 2	EFF	1,2	1,5	No			
Cat 3	PNEU	1,1	0,8	No			
Cat 4	EFF	1,1	0,2	Yes	Yes	Minor	kinking of the wire during insertion
Cat 5	EFF	1,24	1,07	Yes	Yes	Major	middle lung lobe perforation
Cat 6	PNEU	1,14	0,41	Yes	No		
Cat 7	PNEU	1,11	0,45	Yes	Yes	Major	cranial & middle lung lobe perforation
Cat 8	PNEU	1,16	0,92	Yes	No		
Cat 9	EFF	1,24	0,41	Yes	Yes	Major	middle lung lobe perforation
Cat 10	EFF	1,22	0,99	No			
Cat 11	EFF	1,07	0,37	Yes	Yes	Major	caudal lung lobe perforation
Cat 12	EFF	1,11	0,93	Yes	Yes	Major	caudal lung lobe perforation
Cat 13	PNEU	1,17	0,47	No			
Cat 14	PNEU	1,19	0,26	Yes	Yes	Minor	intrathoracic kinking of the drain
Cat 15	PNEU	1,14	0,28	Yes	No		
Cat 16	PNEU	1,15	0,49	No			
Cat 17	PNEU	1,16	0,6	Yes	No		
Cat 18	PNEU	1,18	0,67	No			
Cat 19	EFF	1,2	1,19	Yes	No		
Cat 20	EFF	1,14	0,67	Yes	No		
Cat 21	PNEU	1,18	0,48	Yes	No		
Cat 22	PNEU	1,15	0,64	Yes	No		
Cat 23	EFF	1,14	0,32	Yes	No		
Cat 24	EFF	1,12	0,6	Yes	Yes	Minor	kinking of the wire during insertion

Major complications included lung lobe perforation. Minor complications were kinking of the wire during insertion

Abnormalties were defined as drains situated between lung lobes or doing a loop intrathoracical or diaphragm perforation

*EFF* pleural effusion, *PNEU* pneumothorax

Subsequently, after the placement of the first SBWGTT in the favorable position, patients were repositioned in unfavorable recumbency, and the distance was remeasured. The distribution of intrathoracic contents caused a TWLD with a mean of 0.7 cm in the EFF group (0.2 – 1.55 cm; SD 0.4 cm) and a mean of 0.54 cm in the PNEU group (0.26 – 0.92 cm; SD 0.2 cm) (Table 2). The change in position led to a significant alteration in the TWLD (*p* < 0.001). There was no correlation with the two groups (EFF and PNEU group; *p* = 0.130) and the alteration of the distance due to change in position was similar in both groups.

#### Complications and outcomes

Placement variations within the thoracic cavity were observed in 12 cats, comprising 14/48 tubes and affecting 12/24 cats. These deviations were distinct from the categorized complications (Table 2). Among these anomalies, drains were situated between the lung lobes in 5/14 cases. Notably, in three of these cases, the drain proceeded medially toward the vena cava but without causing perforation. In four instances, out of these 14 thoracostomy drains, the tube formed a loop intrathoracically, and one proceeded between lung lobes.

In a total of eight animals, complications arose during the placement of the chest drain, resulting in a rate of 33.3%. Of these, 6/8 cats were positioned in unfavorable position (75%) for the requested procedure.

Minor complications arose in 3/48 instances. In two cases, they occurred in unfavorable recumbency when the wire couldn’t be advanced forward during insertion due to wire kinking. To solve the problem, a new wire was used. In another cat, the drain showed intrathoracic kinking in favorable position and subsequently proceeded caudally after insertion.

**Fig. 1:**
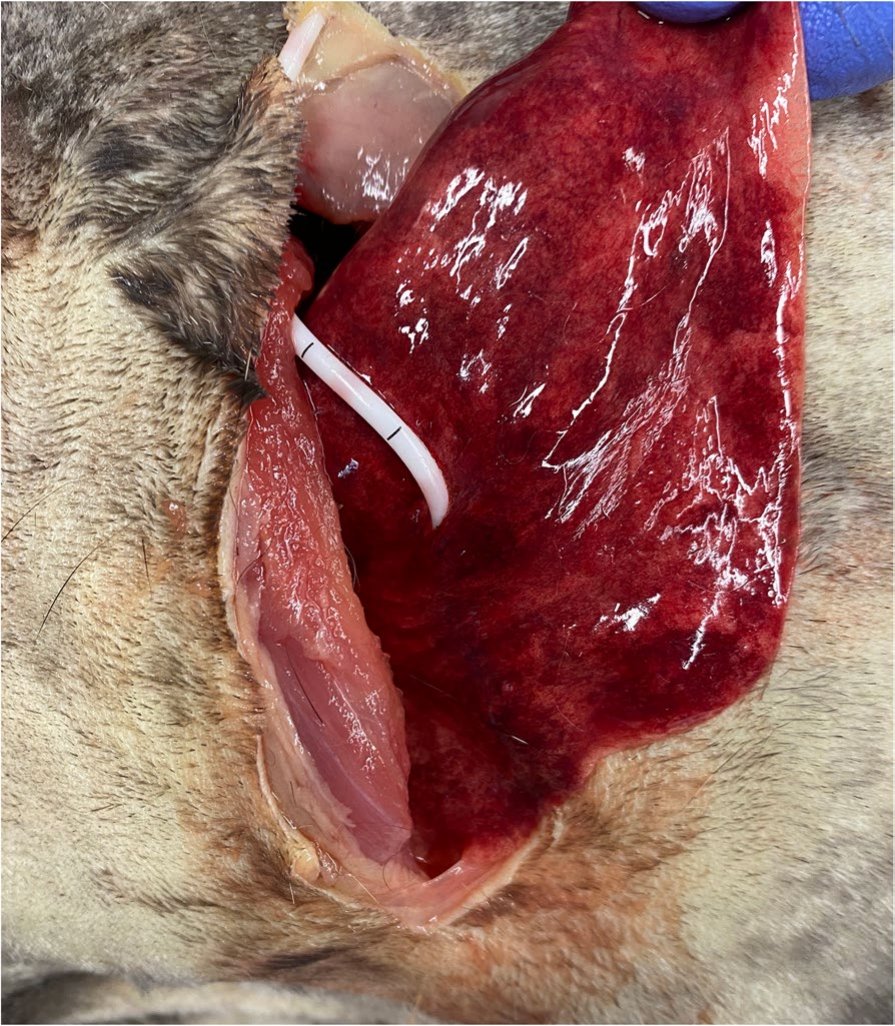
Cadaver no. 7 with the drain located in the middle lung lobe placed in unfavorable recumbency

Major complications were determined in five cats, all of them exhibited lung lobe perforation (Fig. 1). None of these cases involved punctures of the heart, vessels, or other intrathoracic structures. One cadaver showed perforations at multiple sites within the lung lobes, while in another instance, the drain punctured the tip of the middle lung lobe. Additionally, one tube was fully inserted into a lung lobe, and another tube perforated after forming a loop. Four out of five major complications occurred during placement in unfavorable recumbency and the remaining TT was in favorable position. Specifically, 4/5 major complication were identified in the EFF group and 1/5 occurred in the PNEU group.

Furthermore, 6/14 TT resulted in perforation of the diaphragm. All these six cases were in unfavorable recumbent position, with two belonging to the EFF group and four to the PNEU group.

**Fig. 2:**
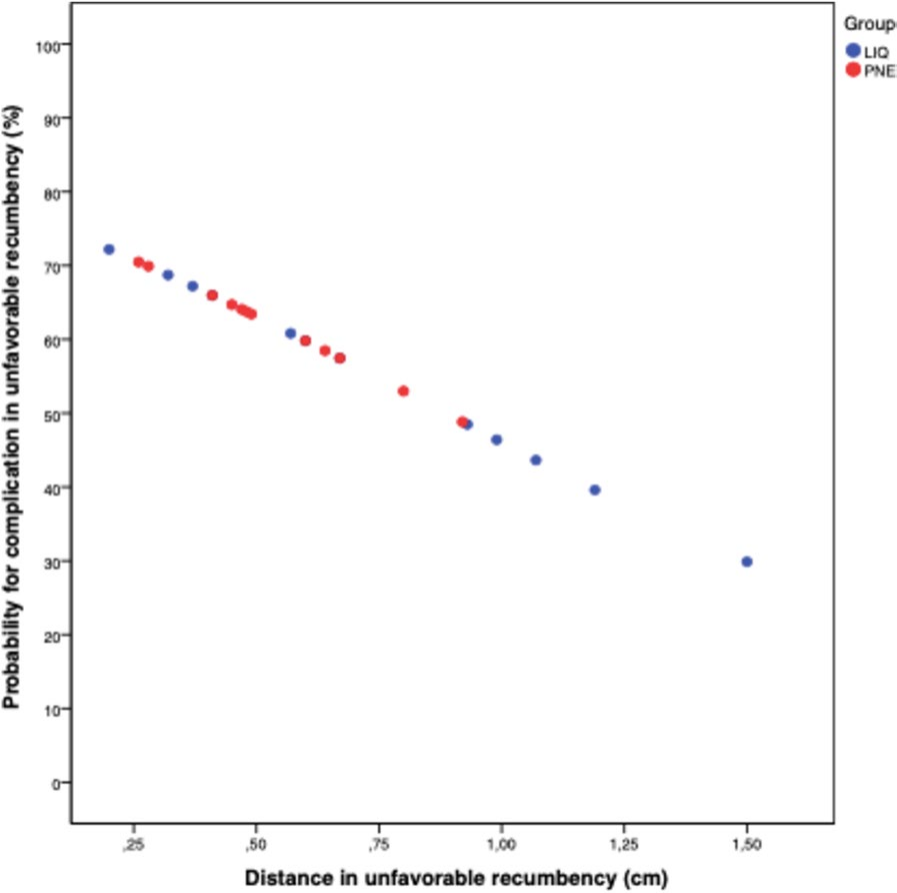
The graph illustrates the risk of complication in unfavorable position: a noticeable reduction in the risk of complications with increasing TWLD can be observed in unfavorable position (from nearly 70% to < 30%)

**Fig. 3:**
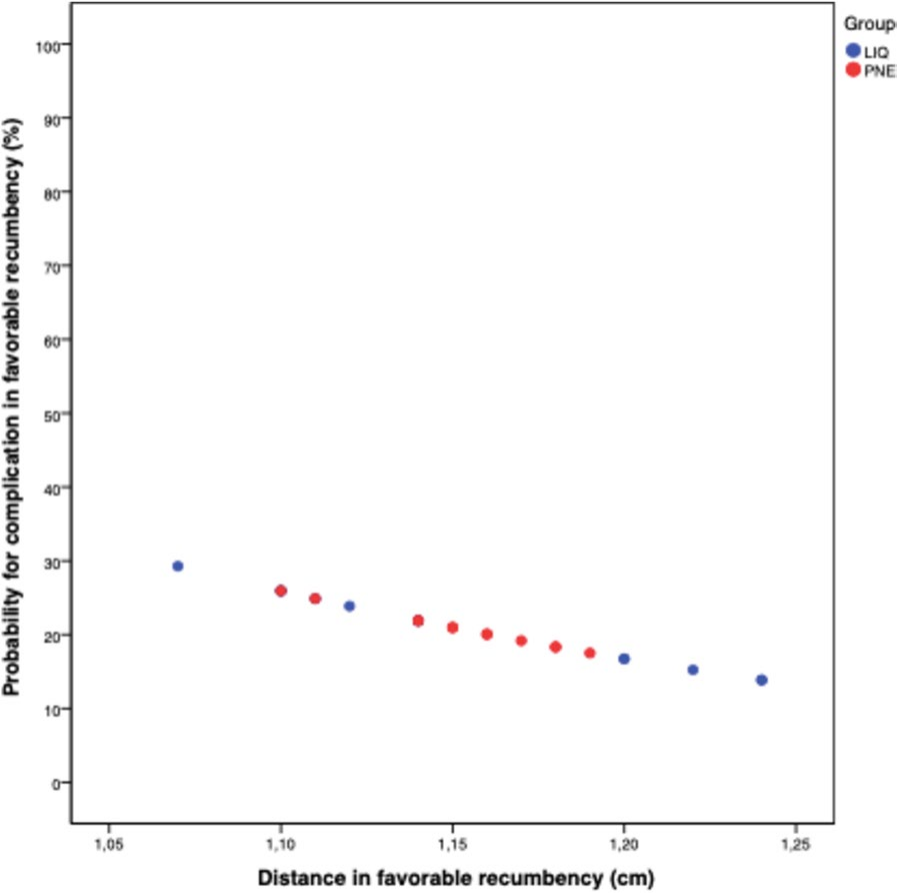
The graph illustrates the risk of complication in favorable position: the risk of complications in favorable position is already significantly lower (at 30%) at a lower distance

In both favorable and unfavorable position, the likelihood of complications decreased with an increase in TWLD, although this was not statistically significant (favorable *p* = 0.631; unfavorable *p* = 0.309). In Fig. 2, a noticeable reduction in the risk of complications with increasing TWLD can be observed in the unfavorable position (from nearly 70% to < 30%), although logistic regression did not yield a statistically significant result. This pattern was also evident in favorable position; however, the risk of complications was already significantly lower (30%) at a lower distance (Fig. 3).

## Discussion

The objective of the study was to investigate the correlation between patients’ recumbency and the occurrence of insertional complications in order to minimize risks and determine factors increasing safety during thoracostomy tube placement with a SBWGTT.

We postulated that, for the treatment of pneumothorax, the small-bore wire-guided tube placement in lateral recumbency would result in fewer insertional complications, as the air would rise to the upper side of the patient, thereby increasing the TWLD. Similarly, for the treatment of fluid-filled thoraces, sternal recumbency would be safer as the fluid would settle down, also increasing the TWLD.

Although SBWGTT is thought to be safer than LBTT, this cadaveric study revealed an overall complication rate of 33.3%, higher than previously reported [11, 17]. These numbers are comparable to a newer retrospective survey from 2022, which reported complications in 32% of dogs and cats with SBWGTT [18]. Similar to other studies, technical issues such as kinking, malpositioning, and accidental removal were common [11, 18]. However, 14.1% of cases in the retrospective survey experienced insertional complications, such as accidental lung lobe perforation [18]. In our study, lung lobe perforation occurred in 20.8% of patients, considerably higher than previously described [11, 17, 18, 33].

Apart from our study and the study of Boullhesen Williams et al. (2022), there are no reports regarding accidental lung lobe perforation with SBWGTT during insertion in the veterinary literature [18]. In human medicine, laceration of the pulmonary tissue is reported in association with the use of traditional large-bore trocar tubes (TRO) in adults and with pigtail-type TT in infants [47–51]. Boullhesen Williams et al. (2022) described lung lobe perforation in two patients (one cat and one dog). These two animals were being treated for pyothorax, and one, which needed lung lobectomy after placing the SBWGTT, had severe adhesions of the lung to the parietal pleura close to the insertion site of the tube. This finding was similar to the human case report where autopsy detected severe pleural adhesions causing lung perforation [18, 47]. Nevertheless, this does not explain our cases with lung lobe laceration.

In a clinical setting, detecting lung lobe perforation caused by TT by diagnostic imaging is challenging and thus may be underestimated [2, 38, 48, 51]. Computed tomography (CT) scans offer precise information about the tube location, indicating whether it is placed within the lung parenchyma [38]. Similar to human medicine, veterinary patients may not always exhibit clinical signs related to lung lobe perforation, and the diagnosis of such perforation is often established during postmortem examinations in humans [18, 47, 49].

Change of the patient’s position from favorable to unfavorable recumbency resulted in a significant decrease in TWLD. Even at the same TWLD, the risk of complication increased by 10% when shifting from a favorable to an unfavorable position, reflecting a higher overall complication rate in the unfavorable position (75%). The likelihood of a complication in the favorable position started at 30%, while in the unfavorable position, it was > 70%. Both probabilities decreased with an increase in TWLD (as shown in Figs. 2 and 3), resulting in a higher risk for complications in the unfavorable position (although logistic regression model couldn’t find statistical significance). A change in position led to a significant decrease in TWLD (*p* < 0.001).

Three of the 48 placed TT (*n* = 3) showed kinking of the wire during insertion. This is described in 14% of the cases in two studies (Valtolina et Adamantos (2009); Boullhesen Williams et al. (2022)) as a minor insertional disadvantage, which could be a consequence due to the wire-guided insertion technique as well as malpositioning. The latter one is outlined as a major complication when malpositioning requires reinsertion [11, 18, 33].

In this study, a cadaver model was created and served as an appropriate model for this trial since negative intrapleural pressure and pulmonary elastic recoil are maintained in the postmortem thorax [52]. Additionally, the utilization of feline cadavers allows the simulation of disease in clinical healthy cats, particularly in a preliminary study regarding complications similar to our trial [12]. Still this study had several limitations inherent to its cadaveric nature.

A study in canine cadavers observed postmortem changes which included gas accumulation in the pleural cavity and lung collapse. These changes were evident through an alveolar pulmonary pattern seen on radiographs, with severe decomposition becoming apparent as early as 24 h after death when stored at an ambient temperature of 22 – 33 °C [53]. Cold storage decelerates this process and improves the quality of the cadavers used for trials [12]. Consequently, this study prompted us to either use feline cadavers immediately after euthanasia or cool them between euthanasia and trial.

In a clinical situation, puncture of the diaphragm may not be important and has not yet been observed to the authors; it likely occurs only in cadavers as they have already undergone *rigor mortis*. Fetzer et al. (2017) have previously documented penetration into the peritoneal cavity in a canine cadaveric study involving four dogs using SBWGTT. They concluded that this phenomenon might be attributed to the distended peritoneal cavity in the cadavers, suggesting that the cranial distension of the diaphragm in these specimens leads to an increased risk of penetrating the diaphragm during placement [12].

To identify the reason for the occurrence in our cats, we tested if it was possible to insert the SBWGTT through the diaphragm into the abdomen. This was only possible in a feline cadaver that was cooled and subsequently warmed, but not in a freshly euthanized (within one hour) cadaver. Since our cadavers were cooled to 9.6 °C degrees and used within 48 h, we consider this complication a sequel to the physical postmortem changes of the diaphragm.

We also conducted tests using only the J-wire, finding it had no effect on the diaphragm. Consequently, we further hypothesize that the wire itself is innocuous, and it is the tip of the drain without the J-wire in it that causes trauma.

Inadvertent placement of the TT into the peritoneal cavity, however, is a described complication in human medicine [54, 55]; none of the recent studies on SBWGTT in veterinary medicine have reported this complication [11, 17, 33]. As previously mentioned, perforation of the diaphragm is unlikely to occur in a clinical patient. It is certain that these drains would be situated in the caudal part of the thorax and not in the cranial pleural cavity, as described in the ideal placement of TT – along the sternum, ventral to the trachea, with the tip of the SBWGTT to the third intercostal space [18, 56]. Another limitation is the lack of statistical significance despite the higher complication rate in unfavorable position. This may be due to a too small sample size. Consequently, although our findings suggest a trend towards more complications in this position, studies with even larger case numbers may be helpful to confirm these findings.

## Conclusion

In conclusion, we found a significant decrease in TWLD when changing from favorable to unfavorable position, resulting in an increased complication rate for patients placed in unfavorable recumbency. We conclude that it is important to adhere to the optimal position for specific conditions: sternal recumbency for pleural effusions and lateral for pneumothorax. Non-compliance with these positions increases the risk of complications, with 75% of patients experiencing complications being positioned in the unfavorable recumbency. Accurate TWLD measurement using ultrasound is essential, as the risk for complications diminishes with increasing distance. We determined a minimum safe distance of 1.1 – 1.2 cm.

However, there is a lack of information regarding major complications associated with patients’ recumbency. Additional prospective trials in canine and feline patients are required to verify whether these results will translate to clinical veterinary patients with naturally occurring disease.

## Data Availability

All data generated or analyzed during this study are included in the published article.

## Abbreviations

BW: Bodyweight
EFF: Pleural effusion
ICS: Intercostal space
JP: Jackson Pratt thoracostomy drain
LBTT: Large-bore thoracostomy tubes
MP: Calculated value of the pilot study
N: Number of individuals
P: Probability value
PIP: Peak inspiratory pressure
PNEU: Pneumothorax
SBTT: Small-bore thoracostomy tubes
SBWGTT: Small-bore wire-guided thoracostomy tubes
TRO: Traditional large-bore trocar tubes
TT: Thoracostomy tubes
TWLD: Lateral thoracic wall to lung distance

## Footnotes

1 Guidewire Inserted Chest Tube; MILA International Inc.®, Erlanger, Florence, Kentucky KY; 20 cm long (8in) polyurethane, multi fenestrated catheter.

2 Vetus 5 exp.®; MINDRAY Animal Medical Technology Co.,LTD.; Shenzhen, China.

3 5 mm Dilating Tip Trocar with Stability Sleeve; Endopath® XCEL™; ETHICON ENDO-SURGERY, LLC; Puerto Rico, USA.
